# Editorial: Unveiling the Impact of Local or Systemic Therapeutic Strategies on the Tumor Microenvironment

**DOI:** 10.3389/fonc.2021.832036

**Published:** 2022-01-25

**Authors:** Yun-Wang Chen, Jia-Hong Jiang, Zhe-Ling Chen, Liu Yang

**Affiliations:** ^1^ Cancer Center, Department of Medical Oncology, Zhejiang Provincial People’s Hospital, Affiliated People’s Hospital, Hangzhou Medical College, Hangzhou, China; ^2^ The Medical College, The Qingdao University, Qingdao, China

**Keywords:** tumor microenvironment, microenvironment-targeting therapy, oncology precision therapy, treatment, immunotherapy

In cancer treatment, chemotherapy and immunotherapy are two important systemic treatments. Yet both of the above treatments are affected by tumor microenvironment (TME). TME is not only the “soil” that supports tumor growth, but also a complex and dynamically changing integrated system, including fibroblasts, immune-inflammatory cells and glial cells, as well as interstitial cells, microvascular system, and infiltrating biomolecules in the adjacent area. TME in a highly immunosuppressive state can significantly attenuate anti-tumoral responses induced by immunotherapy. Some components of TME, such as cancer-associated fibroblasts (CAFs), cytokines, and chemokines secreted by CAFs, can protect tumor cells from the effects of conventional chemotherapy, leading to tumor progression and chemotherapy resistance. Therefore, it is necessary to incorporate microenvironment-targeting therapy into the comprehensive treatment of tumors. In addition, some cells or molecules that make up TME can also be used as potential indicators to predict cancer prognosis and evaluate the efficacy of chemotherapy or immunotherapy.

TME manifests as an acidic microenvironment, characterized by hypoxia, angiogenesis, inflammation, and immunosuppression. Tumor cells are able to overcome the above TME hardships during the growth phase to achieve a stronger growth advantage than normal cells. The tumor microenvironment-targeting therapies aimed at its complex biological characteristics and various components of TME have achieved good therapeutic outcomes. Hypoxia-activated prodrugs have been widely investigated for targeting hypoxic tumor cells. Canakinumab, a selective IL-1 β inhibitor, can inhibit the related inflammatory response in TME and reduce immunosuppression. According to the CANTOS study, compared with the placebo group, canakinumab treatment could significantly reduce the incidence rates and mortality of lung cancer (1). The immunosuppressive environment in TME can attenuate the antitumor activity of natural killer (NK) cells. Metabolic flexibility determines NK cell functional fate in TME (2). Compared with the original NK cells, the expanded NK cells with complete metabolic flexibility have stronger tumor killing in TME. Except for non-cellular components, tumor-associated macrophages (TAMs), Tregs, myeloid-derived suppressor cells (MDSCs), and other cellular components with immunosuppressive effects in TME play an important role in tumor proliferation, migration, invasion, metastasis, and chemoresistance. Targeted blocking of the above components could kill tumors and reverse chemotherapy resistance. TME provides the “soil” for tumor to “grow”, which leads to the production of nonsynonymous mutation of tumor cells, namely neoantigens. Neoantigens are also the high-quality targets for microenvironment-targeting therapies. Individualized neoantigen peptide vaccines can induce sustained T cell responses (3). At the same time, microenvironment-targeting therapies may also have synergistic effects with routine chemotherapy, immunotherapy, and other targeted treatment strategies. In addition to tumor microenvironment targeting therapies, it also makes sense to predict chemotherapy and immunotherapy by TME related indicators. Analyzing the changes of immune status before and after anti-tumor treatment or developing a visualization tool of personalized treatment based on genomics are important elements and tools for precision tumor therapy (4). This Research Topic includes three review articles and five original research articles on TME.

Wang et al. summarized the TME of esophageal cancer (EC) and the latest progress in microenvironment-targeting therapies. This paper summarized that some stromal components and important signaling pathways in TME played an important role in the evolution of EC. It also pointed out that suppressing inflammation in TME, anti-angiogenesis, and improving the hypoxic microenvironment could achieve the aim of preventing and treating tumors. Guo et al. have developed a novel NK cell expansion method utilizing OX40L armed NK-92 cell with secreting neoleukin-2/15 (Neo-2/15). These cells have high cytotoxicity against Raji cells and against HepG2 (liver cancer), A427 (lung cancer), and CAVO3 (ovarian cancer) *in vivo*. The complex TME of solid tumor can block the infiltration of NK cells, and the NK92-Neo2/15-OX40L expanded NK cells have stronger infiltration ability and antitumor activity. Cao et al. summarized the characteristics and functions of some key cellular components of TME. On this basis, this article explored the potential treatment strategies of targeted therapy for the above factors in order to eliminate drug resistance and increase therapeutic efficacy. Zhang et al. identified and analyzed multiple neoantigens by whole-exome sequencing of tumor specimens from three patients with non-small cell lung cancer (NSCLC). Neoantigens from three patients successfully induced neoantigen-reactive T cells (NRTs), which showed satisfactory results and anti-tumor effects, as demonstrated with mouse model. The treatment method aiming at the specific antigen produced by tumor cells can efficiently control and even cure the tumor, so as to achieve the purpose of oncology precision therapy. Turinetto et al. summarized the effects of poly (ADP-ribose) polymerase inhibitors (PARPi) on TME in ovarian cancer from the perspectives of hypoxia, cGAS-cGAMP-STING pathway, and upregulation of PDL-1. It shows that PARP inhibitors plus other target therapies or immunotherapy can improve the prognosis by influencing the immune system and the TME.

Wang et al. collected immunological indexes before and after neoadjuvant chemotherapy in 262 breast cancer patients and selected five of these indexes for analysis to form a prediction model, named NeoAdjuvant Therapy Immune Model (NATIM). Three indexes - CD4+/CD8+ T cell ratio (a/b), CD3+CD8+ cytotoxic T cell percent (a/b), and lymphosum of T, B, and NK cells (a/b) - were thought to be effective predictors of neoadjuvant chemotherapy. Using bioinformatics and cytological tests Aili et al. confirmed that knockdown of PBRM1 was associated with the reduction of CD4 T cells in clear cell renal cell carcinoma (ccRCC) TME and anti-PD-1 immunotherapy can increase the infiltration of T cells in both PBRM1 high and PBRM1 low tumors, but to different degrees. High PBRM1 expression levels imply more T cells in TME and PBRM1 expression levels can influence and predict the efficacy of anti-PD-1 immunotherapy. Zhang et al. performed comprehensive bioinformatics analysis of the published datasets of head and neck squamous cell carcinoma (HNSCC) and proposed a novel molecular subtype classification strategy that can predict disease prognosis and guide treatment allocation.

Microenvironment-targeting therapies improve the therapeutic efficacy of existing tumor methods and reverse drug resistance. Analyzing various immunological indexes in TME and classifying molecular subtypes including TME can guide treatment allocation and predict disease prognosis, so as to achieve the purpose of personalized oncology precision therapy. However, there are still many problems to be solved about the clinical guidance of the TME in the future. Future research does not only need more in-depth excavation of the ingredients in the TME but also needs to develop an accurate treatment for microenvironment targets. With this Research Topic, the editors hope to inspire subsequent research for TME and the further development of microenvironment-targeting therapies.

## Author Contributions

LY and Z-LC contributed to the conception of the article. Y-WC integrated all information and wrote the manuscript. LY and Z-LC provided critical guidance, revisions for J-HJ throughout the writing process. Y-WC, J-HJ, and LY compiled information and revised the manuscript. All authors read and approved the final manuscript.

## Conflict of Interest

The authors declare that the research was conducted in the absence of any commercial or financial relationships that could be construed as a potential conflict of interest.

## Publisher’s Note

All claims expressed in this article are solely those of the authors and do not necessarily represent those of their affiliated organizations, or those of the publisher, the editors and the reviewers. Any product that may be evaluated in this article, or claim that may be made by its manufacturer, is not guaranteed or endorsed by the publisher.
